# The role of the brown adipose tissue in β3-adrenergic receptor activation-induced sleep, metabolic and feeding responses

**DOI:** 10.1038/s41598-017-01047-1

**Published:** 2017-04-19

**Authors:** Éva Szentirmai, Levente Kapás

**Affiliations:** 1 Elson S. Floyd College of Medicine, Department of Biomedical Sciences, Washington State University, Spokane, WA USA; 2 Sleep and Performance Research Center, Washington State University, Spokane, WA USA

## Abstract

Brown adipose tissue (BAT) is regulated by the sympathetic nervous system via β3-adrenergic receptors (β3-AR). Here we tested the hypothesis that pharmacological stimulation of β3-ARs leads to increased sleep in mice and if this change is BAT dependent. In wild-type (WT) animals, administration of CL-316,243, a selective β3-AR agonist, induced significant increases in non-rapid-eye movement sleep ﻿(NREMS) lasting for 4–10 h. Simultaneously, electroencephalographic slow-wave activity (SWA) was significantly decreased and body temperature was increased with a delay of 5–6 h. In uncoupling protein 1 (UCP-1) knockout mice, the middle and highest doses of the β3-AR agonist increased sleep and suppressed SWA, however, these effects were significantly attenuated and shorter-lasting as compared to WT animals. To determine if somnogenic signals arising from BAT in response to β3-AR stimulation are mediated by the sensory afferents of BAT, we tested the effects of CL-316,243 in mice with the chemical deafferentation of the intra-scapular BAT pads. Sleep responses to CL-316,243 were attenuated by ~50% in intra-BAT capsaicin-treated mice. Present findings indicate that the activation of BAT via β3-AR leads to increased sleep in mice and that this effect is dependent on the presence of UCP-1 protein and sleep responses require the intact sensory innervation of BAT.

## Introduction

Adipose tissue plays a central role in the interplay between nutrition, energy balance, and health. Two distinct types of adipose tissue with fundamentally different functions exist – brown and white adipose tissue^1^. The tightly regulated balance between the activities of the two fat tissues is critical in maintaining metabolic homeostasis. White adipose tissue (WAT) stores excess energy in the form of fat, whereas brown adipose tissue (BAT) dissipates energy as heat. BAT was initially believed to play a role in hibernating animals, but its presence in non-hibernating animals and humans suggests a wider array of functions. The largest BAT depot in rodents is located in the interscapular region (iBAT). Other depots are present in the axillary, cervical and paravertebral regions as well as around thoracic and abdominal viscera.

BAT controls energy balance via regulated (adaptive) heat production. The thermogenic property of BAT is conferred by the tissue-specific presence of uncoupling protein 1 (UCP-1). BAT thermogenesis is under the control of the sympathetic nervous system^2^. Norepinephrine, released from the nerve terminals, facilitates lipolysis in BAT by acting on β3-adrenergic receptors (β3-ARs)^3^.

β3-ARs are expressed predominantly by white and brown adipocytes, and smooth muscle cells of various organs^4–6^. Selective, pharmacological activation of β3-ARs has been shown to have profound effects on adipose tissue morphology and metabolism. Administration of CL-316,243, a potent and highly selective β3-AR agonist^7^, leads to marked increases in thermogenesis by BAT, lipolysis in WAT, and an acute decrease in food consumption^8–10^. Long-term treatment of obese rodents with β3-AR agonists reduces fat stores and improves obesity-induced insulin resistance as well as facilitates the appearance of brown adipocytes in WAT tissue (“browning”)^11, 12^. β3-AR knockout (KO) mice are completely resistant to the effects of CL-316,243 indicating that these effects are mediated exclusively by β3-AR^8^.

Previous studies in our laboratory showed a role for BAT in sleep regulation^13^. Sleep loss stimulates BAT thermogenesis as shown by increased BAT temperature and 6-fold increases in UCP-1 mRNA. Recovery sleep after sleep deprivation is significantly attenuated in UCP-1 KO mice indicating that BAT heat production is required for rebound sleep after sleep loss.

BAT is supplied by rich afferent innervation^14–16^. Anterograde transneuronal viral tracing showed that sensory information reaches the brain via ascending spinal pathways in the anterolateral system. The afferents are sensitive to changes in local BAT temperature^17^, thus they are well positioned to convey temperature-related signals associated with BAT activation to brain sleep centers. Hypothalamic nuclei, periaqueductal gray, parabrachial nuclei, raphe nuclei, and the nucleus of the solitary tract are main elements of the BAT central sensory circuits^14, 16^. Sensory denervation of BAT by capsaicin suppressed rebound sleep increases after sleep deprivation by ~50% in mice^13^. These results indicate that sleep-promoting signals arising from BAT in response to sleep loss are mediated by small-diameter sensory afferents.

In the present study we tested the effects of acute, pharmacological stimulation of β3-ARs on sleep, body temperature and metabolic parameters in mice. We report that CL-316,243 induced immediate and long-lasting increases in non-rapid-eye movement sleep (NREMS) as well as increases in body temperature in wild-type (WT) mice. To establish the role of BAT in β3-AR stimulation-induced sleep responses, we also studied the sleep and body temperature-modulating effects of the β3-AR agonist in UCP-1 KO mice and in mice after the sensory deafferentation of the iBAT. We report that CL-316,243-induced sleep requires the intact thermogenic activity of BAT, and sleep-promoting signals that arise from activated BAT require the intact sensory innervation of the tissue to enhance sleep.

## Results

### The effect of CL-316,243 on sleep-wake activity in WT and UCP-1 KO mice

The behavioral effects of CL-316,243 were not quantified, but on-line video observation of the animals did not reveal any gross behavioral effects. The animals moved around in their cages after the treatment and most, especially in the WT group, assumed typical sleeping position within 10–20 min.

**Figure 1:**
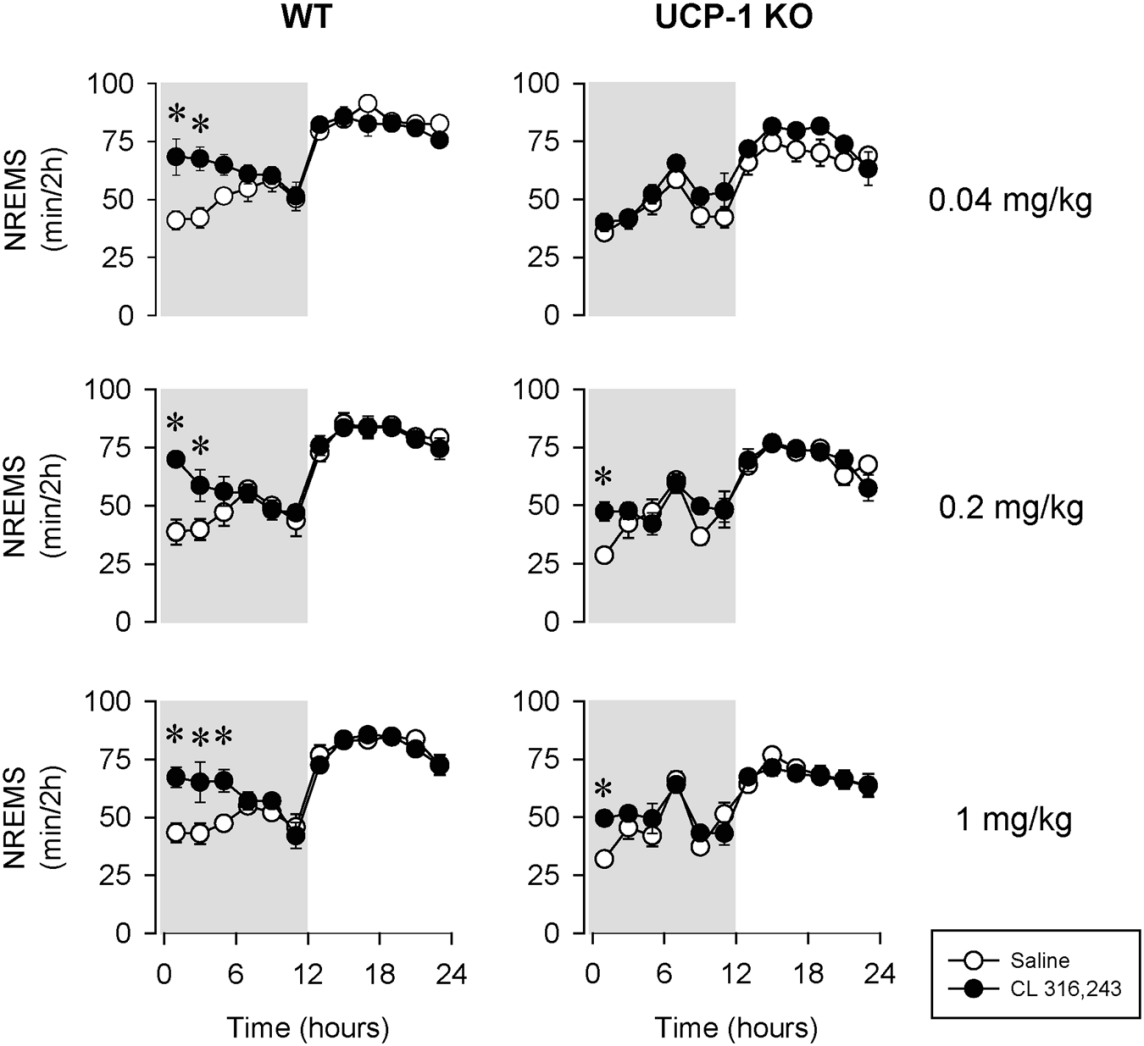
The effects of the selective β3-adrenergic receptor (β3-AR) agonist CL-316,243 (black symbols) on non-rapid-eye movement sleep (NREMS) in wild-type (WT; left panels) and uncoupling protein-1 knockout (UCP-1 KO; right panels) mice. On the baseline day saline (open symbols) was administered subcutaneously 10–15 min before the onset of the dark phase. On the experimental day, one dose of CL-316,243 (0.04, 0.2 or 1 mg/kg; top, middle and bottom panels, respectively) was injected prior dark onset. Asterisk (*) denotes significant difference from baseline, p < 0.05. Error bars: standard error. Grey areas represent the dark phase.

CL-316,243 induced immediate increases in NREMS in WT mice (Fig. 1, Table 1). NREMS was significantly elevated for 4 hours after the 0.04 and 0.2 mg/kg doses of CL-316,243 by 60 and 65%, respectively. The increases lasted for 6 hours after the 1 mg/kg dose. After 4–6 hours, NREMS amounts returned to baseline.

**Table 1:** Effects of CL-316,243 on NREMS in WT and UCP-1 KO mice – statistical results.

0.04 mg/kg CL-316,243- NREMS	df	F	p
Genotype	1,11	20.9	<0.05
Treatment	1,11	7.2	<0.05
Treatment × genotype	1,11	0.5	n.s
Time	11,121	41.3	<0.05
Time × genotype	11,121	1.3	n.s
Treatment × time	11,121	2.0	<0.05
Treatment × time × genotype	11,121	2.9	<0.05
**0.2 mg/kg CL-316,243 - NREMS**	**df**	**F**	**p**
Genotype	1,11	19.8	<0.05
Treatment	1,11	10.5	<0.05
Treatment × genotype	1,11	0.8	n.s
Time	11,121	39.9	<0.05
Time × genotype	11,121	1.8	n.s
Treatment × time	11,121	4.8	<0.05
Treatment × time × genotype	11,121	1.3	n.s
**1 mg/kg CL-316,243 - NREMS**	**df**	**F**	**p**
Genotype	1,10	33.8	<0.05
Treatment	1,10	13.4	<0.05
Treatment × genotype	1,10	3.3	n.s
Time	10,110	38.5	<0.05
Time × genotype	10,110	3.5	<0.05
Treatment × time	10,110	6.8	<0.05
Treatment × time × genotype	10,110	1.0	n.s

**Figure 2:**
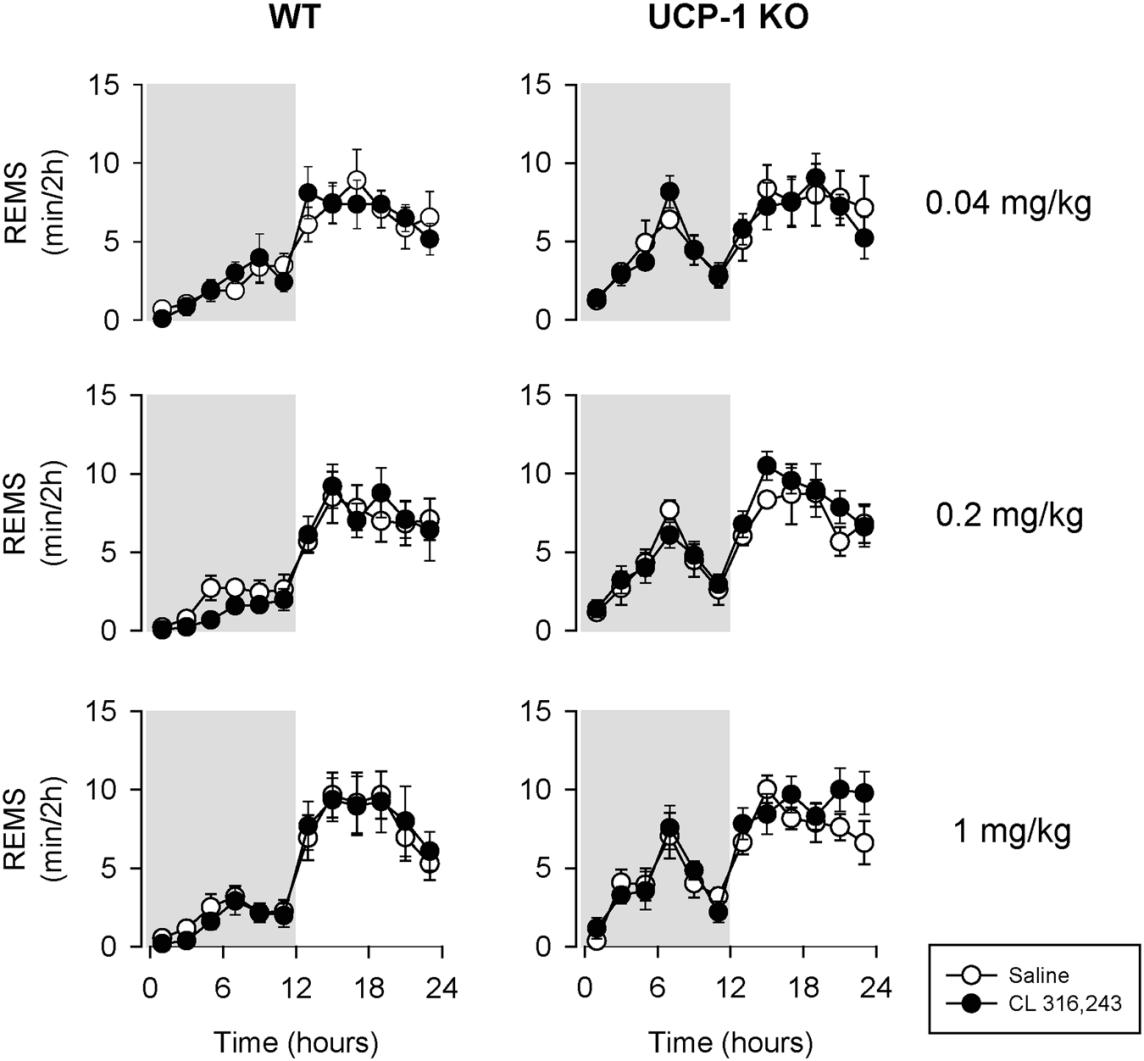
The effects of the selective β3-AR agonist CL-316,243 on rapid-eye movement sleep (REMS) in WT (left panels) and UCP-1 KO (right panels) mice. See Fig. 1 for details.

In UCP-1 KO mice, the lowest, 0.04 mg/kg, dose of CL-316,243 failed to induce any changes in NREMS. The 0.2 and 1 mg/kg doses of CL-316,243 were followed by significant increases in NREMS, however, these changes were significantly attenuated and shorter-lasting compared to control mice. In the first 4 hours after 0.2 mg/kg CL-316,243 injection, NREMS increase in UCP-1 KO mice was significantly, by ~50% less than in controls (23.8 ± 8.5 min in UCP-1 KO mice vs. 50.2 ± 8.0 min in WT, p < 0.05). Similarly, 1 mg/kg CL-316,243 was followed by a significantly attenuated NREMS increase in UCP-1 KO mice (31.1 ± 10.5 min in UCP-1 KO mice vs. 64.2 ± 8.7 min in WT, p < 0.05). While the effects of CL-316,243 on NREMS were significantly attenuated, they still showed dose-dependency (one-way ANOVA on NREMS amounts in h 1–6 across all doses: F(3, 20) = 3.59, p < 0.05). CL-316,243 had no effect on the amounts of rapid-eye movement sleep (REMS) in WT or UCP-1 KO mice (Fig. 2, Table 2).

**Table 2:** Effects of CL-316,243 on REMS in WT and UCP-1 KO mice – statistical results.

0.04 mg/kg CL-316,243 - REMS	df	F	p
Genotype	1,11	1.0	n.s.
Treatment	1,11	0.04	n.s.
Treatment × genotype	1,11	0.03	n.s.
Time	11,121	19.1	<0.05
Time × genotype	11,121	2.2	<0.05
Treatment × time	11,121	1.5	n.s.
Treatment × time × genotype	11,121	0.5	n.s.
**0.2 mg/kg CL-316,243 - REMS**	**df**	**F**	**p**
Genotype	1,11	3.0	n.s.
Treatment	1,11	0.2	n.s.
Treatment × genotype	1,11	6.7	<0.05
Time	11,121	32.9	<0.05
Time × genotype	11,121	2.0	<0.05
Treatment × time	11,121	1.3	n.s.
Treatment × time × genotype	11,121	0.4	n.s.
**1 mg/kg CL-316,243 - REMS**	**df**	**F**	**p**
Genotype	1,10	1.5	n.s.
Treatment	1,10	1.7	n.s.
Treatment × genotype	1,10	3.0	n.s.
Time	10,110	36.4	<0.05
Time × genotype	10,110	2.4	<0.05
Treatment × time	10,110	1.3	n.s.
Treatment × time × genotype	10,110	0.3	n.s.

**Figure 3:**
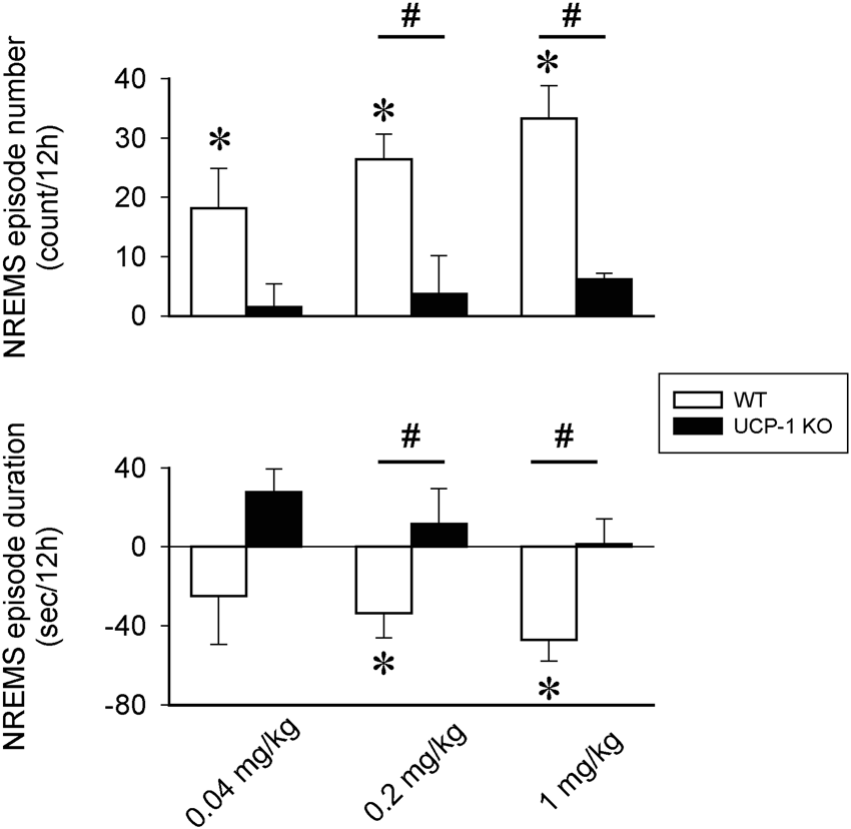
The effects the selective β3-AR agonist CL-316,243 on the number and average duration of NREMS episodes in the first 12 h after treatment in WT (white bars) and UCP-1 KO mice (black bars). Data represent differences from baseline (vehicle). Error bars: standard error. Asterisk (*) denotes significant difference from baseline, # denotes significant difference between genotypes, p < 0.05.

In WT animals, the increases in NREMS time were accompanied by increased number of NREMS episodes after all three doses of CL-316,243, and decreased average duration of the episodes after the middle and high doses (Fig. 3). In UCP-1 KO mice, there was a tendency towards increased NREMS episode numbers and durations, but the changes were not significant. CL-316,243 treatment did not affect REMS episode numbers or durations (data not shown).

**Figure 4:**
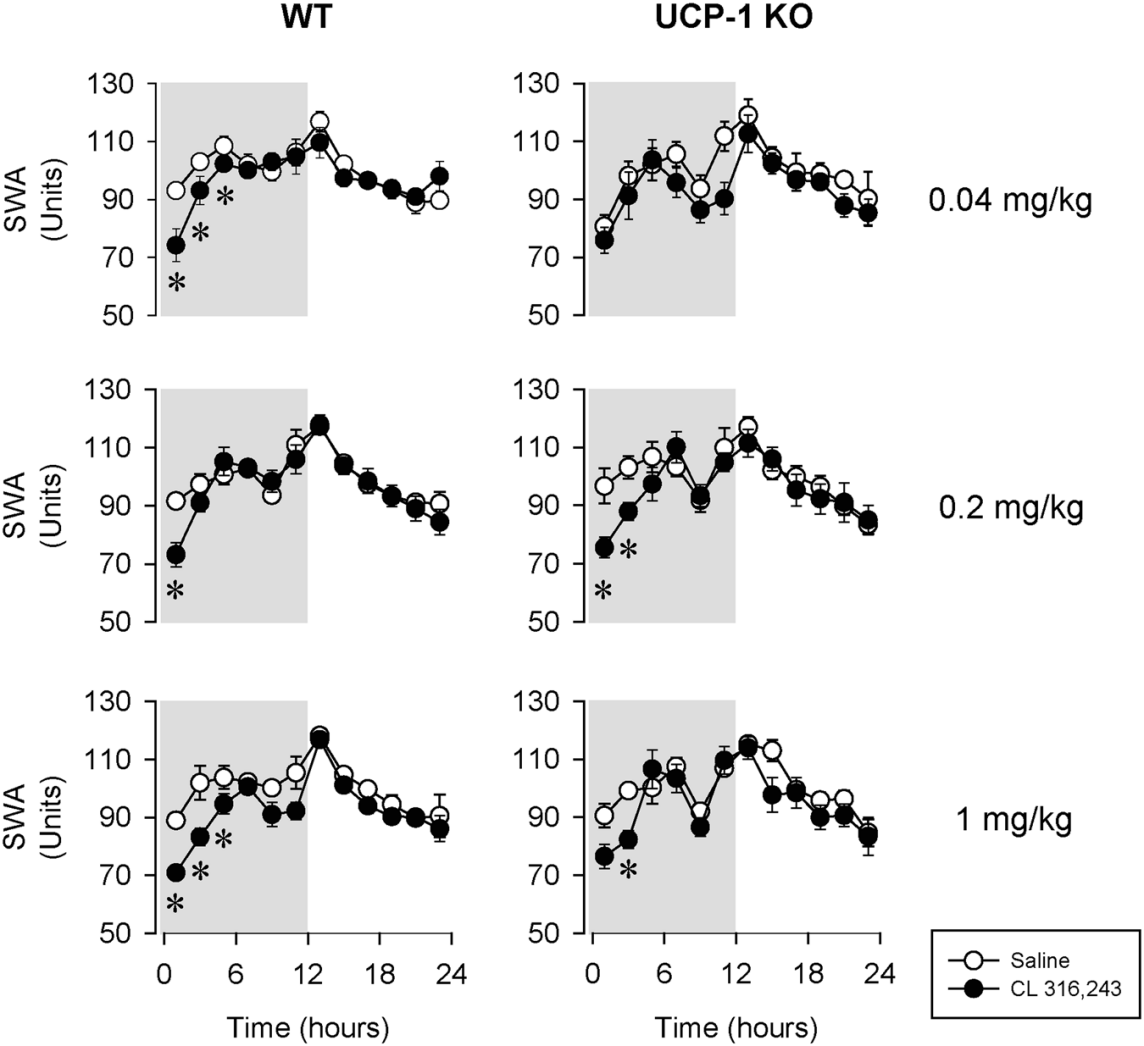
The effects of the selective β3-AR agonist, CL-316,243 on electroencephalographic slow-wave activity (EEG SWA) in WT (left panels) and UCP-1 KO (right panels) mice. See Fig. 1 for details.

**Figure 5:**
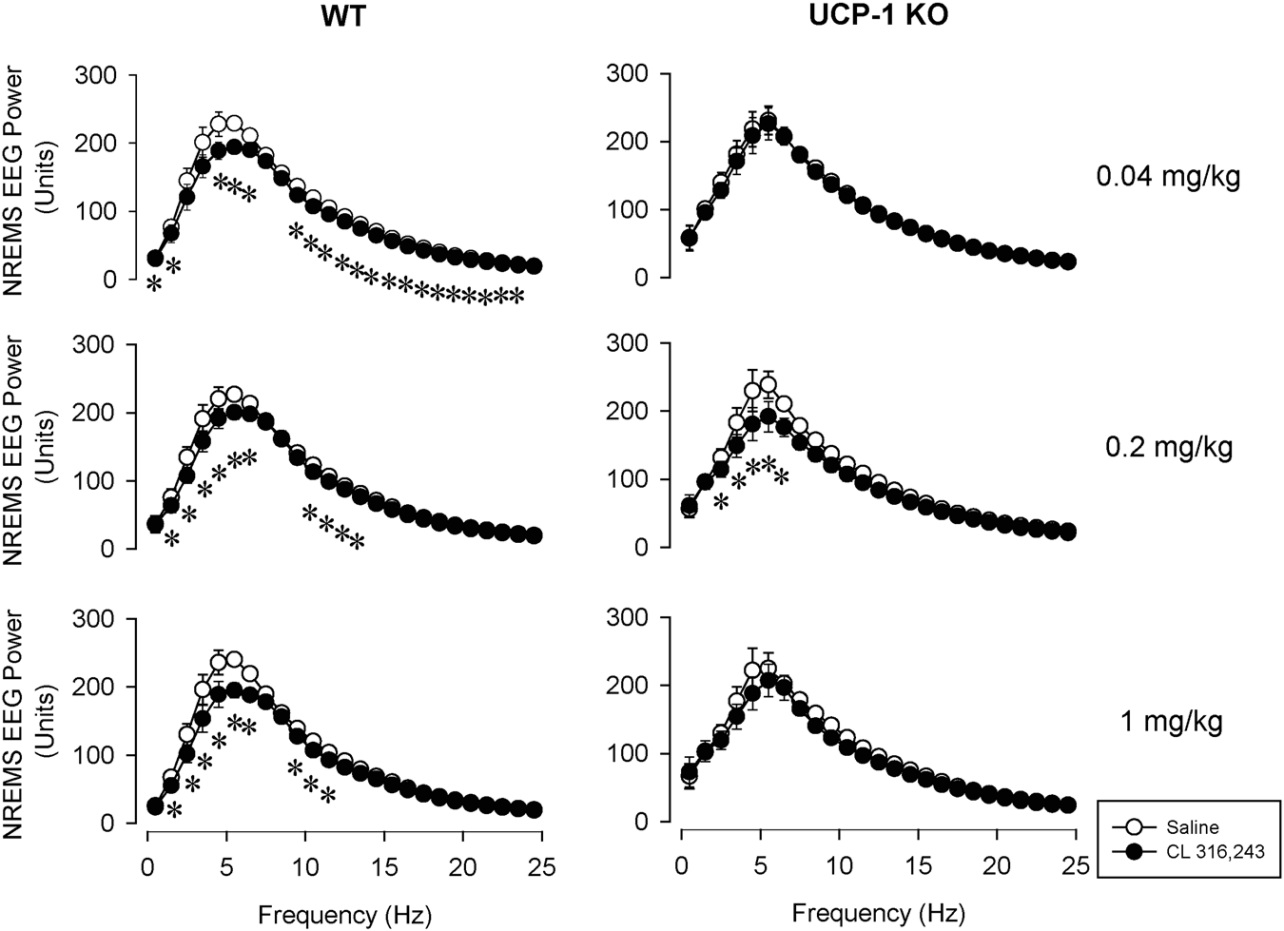
The effects of the selective β3-AR agonist, CL-316,243 on EEG power in the 0.5–25 Hz range in WT (left panels) and UCP-1 KO (right panels) mice. Power values represent the average in the first 6 h after the treatments. See Fig. 1 for details.

Electroencephalographic (EEG) slow-wave activity (SWA) was significantly reduced for 2–6 h after all three doses of CL-316,243 in WT mice (Fig. 4, Table 3). The lowest 0.04 mg/kg CL-316,243 failed to induce any changes on EEG SWA in UCP-1 KO mice. The middle and high doses of CL-316,243 were followed by slight but significant decreases in EEG SWA. Analysis of the higher EEG frequencies (4.5–25 Hz) revealed that the suppression of EEG power was not restricted to the slow-wave band (Fig. 5, Table 4).

**Table 3:** Effects of CL-316,243 on EEG SWA in WT and UCP-1 KO mice – statistical results.

0.04 mg/kg CL-316,243 – EEG SWA	df	F	p
Genotype	1,11	1.4	n.s
Treatment	1,11	11.5	<0.05
Treatment × genotype	1,11	1.4	n.s
Time	11,121	11.0	<0.05
Time × genotype	11,121	0.8	n.s
Treatment × time	11,121	1.5	n.s.
Treatment × time × genotype	11,121	2.0	<0.05
**0.2 mg/kg CL-316,243 - EEG SWA**	**df**	**F**	**p**
Genotype	1,11	0.1	n.s
Treatment	1,11	4.4	n.s
Treatment × genotype	1,11	0.1	n.s
Time	11,121	19.0	<0.05
Time × genotype	11,121	0.5	n.s
Treatment × time	11,121	4.0	<0.05
Treatment × time × genotype	11,121	1.5	n.s.
**1 mg/kg CL-316,243 - EEG SWA**	**df**	**F**	**p**
Genotype	1,10	0.4	n.s.
Treatment	1,10	12.0	<0.05
Treatment × genotype	1,10	0.3	n.s.
Time	10,110	22.3	<0.05
Time × genotype	10,110	1.2	n.s.
Treatment × time	10,110	3.5	<0.05
Treatment × time × genotype	10,110	1.8	n.s.

**Table 4:** Effects of CL-316,243 on the NREMS EEG power spectrum averaged for the first 6 h in WT and UCP-1 KO mice – statistical results.

0.04 mg/kg CL – EEG power 0.5–25 Hz	df	F	p
Genotype	1,11	2.8	n.s
Treatment	1,11	12.1	<0.05
Treatment × genotype	1,11	0.3	n.s
Frequency	24,264	87.5	<0.05
Frequency × genotype	24,264	0.4	n.s
Treatment × Frequency	24,264	9.5	<0.05
Treatment × Frequency × genotype	24,264	1.5	<0.05
**0.2 mg/kg CL - EEG power 0.5–25 Hz**	**df**	**F**	**p**
Genotype	1,11	0.1	n.s
Treatment	1,11	20.3	<0.05
Treatment × genotype	1,11	1.3	n.s
Frequency	24,264	98.7	<0.05
Frequency × genotype	24,264	0.6	n.s
Treatment × Frequency	24,264	12.6	<0.05
Treatment × Frequency × genotype	24,264	2.2	<0.05
**1 mg/kg CL - EEG power 0.5–25 Hz**	**df**	**F**	**p**
Genotype	1,10	2.8	n.s
Treatment	1,10	11.3	<0.05
Treatment × genotype	1,10	0.4	n.s
Frequency	24,240	91.7	<0.05
Frequency × genotype	24,240	0.9	n.s
Treatment × Frequency	24,240	10.3	<0.05
Treatment × Frequency × genotype	24,240	2.2	<0.05

**Figure 6:**
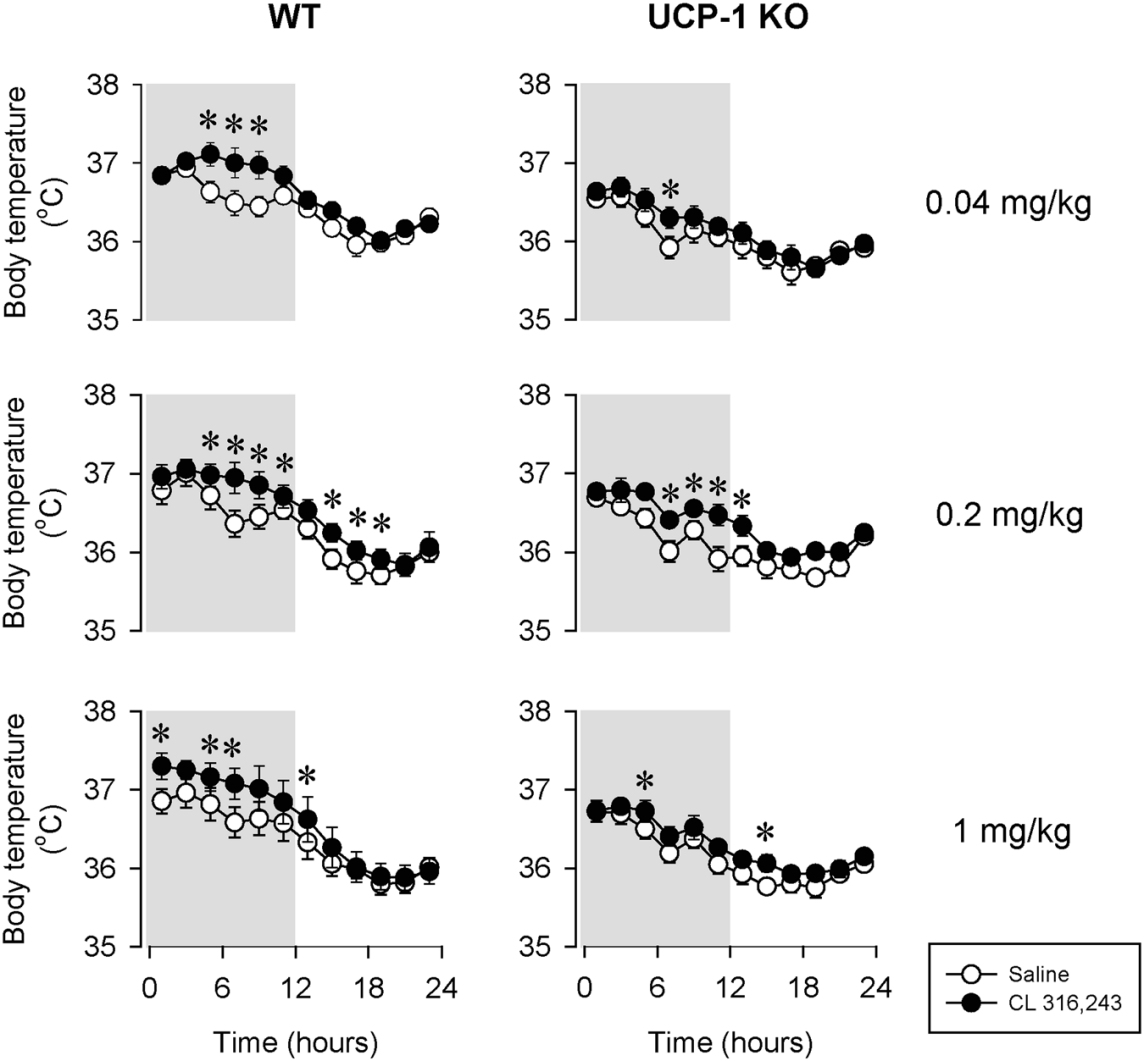
The effects of the selective β3-AR agonist CL-316,243 on body temperature in WT (left panels) and UCP-1 KO (right panels) mice. See Fig. 1 for details.

Activation of β3-ARs stimulates thermogenesis (Fig. 6, Table 5). Core body temperature rose with a delay of 5 h after the 0.04 and 0.2 mg/kg doses of CL-316,243 in WT mice. The highest dose of CL-316,243 increased body temperature immediately after injection. Compared to controls, increases in body temperature were more delayed and significantly attenuated in UCP-1 KO mice after the 0.04 and 1 mg/kg doses (Student’s t-test on 12-h temperature averages; difference from baseline averaged for the first 12 h in WT: 0.36 ± 0.07 °C, 0.28 ± 0.05 °C, 0.37 ± 0.11 °C and in UCP-1 KO: 0.18 ± 0.04 °C, 0.31 ± 0.12 °C, 0.15 ± 0.04 °C after the 0.04, 0.2 and 1 mg/kg doses, respectively).

**Table 5:** Effects of CL-316,243 on body temperature in WT and UCP-1 KO mice – statistical results.

0.04 mg/kg CL-316,243 – Body Temperature	df	F	p
Genotype	1,11	8.1	<0.05
Treatment	1,11	24.1	<0.05
Treatment × genotype	1,11	1.5	n.s.
Time	11,121	55.9	<0.05
Time × genotype	11,121	1.6	n.s.
Treatment × time	11,121	3.5	<0.05
Treatment × time × genotype	11,121	1.1	n.s.
**0.2 mg/kg CL-316,243 – Body Temperature**	**df**	**F**	**p**
Genotype	1,11	0.8	n.s.
Treatment	1,11	17.9	<0.05
Treatment × genotype	1,11	0.0	n.s.
Time	11,121	79.8	<0.05
Time × genotype	11,121	6.5	<0.05
Treatment × time	11,121	3.5	<0.05
Treatment × time × genotype	11,121	1.9	<0.05
**1 mg/kg CL-316,243 – Body Temperature**	**df**	**F**	**p**
Genotype	1,10	2.3	n.s.
Treatment	1,10	16.1	<0.05
Treatment × genotype	1,10	0.3	n.s.
Time	10,110	61.9	<0.05
Time × genotype	10,110	5.9	<0.05
Treatment × time	10,110	2.4	<0.05
Treatment × time × genotype	10,110	2.6	<0.05

**Figure 7:**
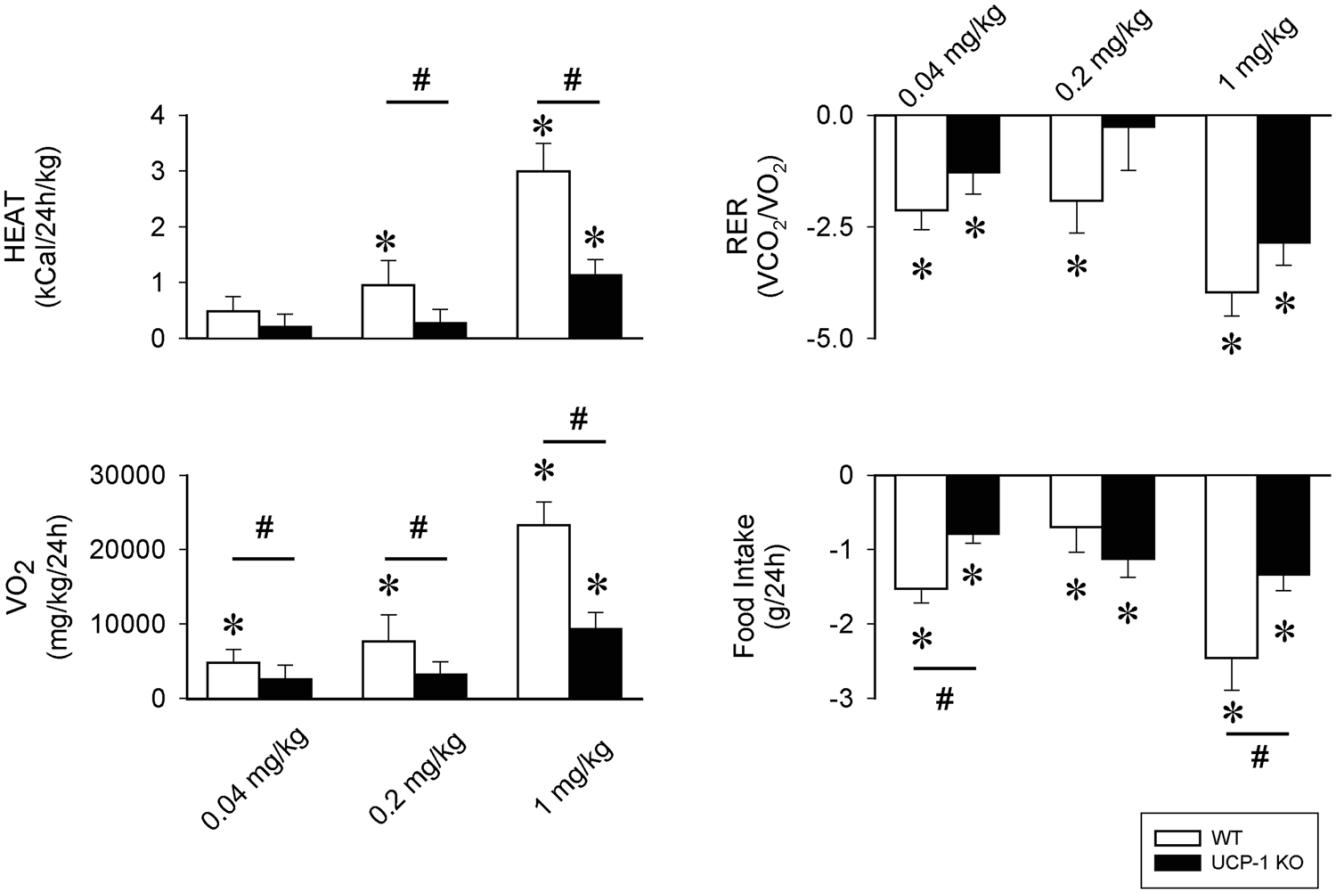
The effects of the selective β3-AR agonist CL-316,243 on heat production (HEAT), O_2_ consumption (VO_2_), respiratory exchange ratio (RER) and 24-h food intake in WT (white bars) and UCP-1 KO mice (black bars). Data represent differences from baseline (vehicle). Error bars: standard error. Asterisk (*) denotes significant difference from baseline, # denotes significant difference between genotypes, p < 0.05.

All three doses of CL-316,243 induced increases in heat production, oxygen uptake ﻿(VO_2_) and suppression of respiratory exchange ratio (RER) and feeding in both genotypes (Fig. 7, Tables 6, 7, 8 and 9). Changes in heat production and VO_2_ in UCP-1 KO mice, however, were significantly attenuated compared to WTs. There was a tendency towards attenuated RER response to CL-316,243 in the KO animals, but the differences between the two genotypes did not reach the level of significance. Decreases in 24-h food intake was also significantly different between WT and UCP-1 KO mice with KO mice showing smaller decreases after the 0.04 and 1 mg/kg doses of CL-316,243.

**Table 6:** Effects of CL-316,243 on heat production in WT and UCP-1 KO mice – statistical results.

0.04 mg/kg CL-316,243 - HEAT	df	F	p
Genotype	1,14	12.7	<0.05
Treatment	1,14	3.6	n.s.
Treatment × genotype	1,14	0.5	n.s.
**0.2 mg/kg CL-316,243 - HEAT**	**df**	**F**	**p**
Genotype	1,14	6.9	<0.05
Treatment	1,14	23.9	<0.05
Treatment × genotype	1,14	13.5	<0.05
**1 mg/kg CL-316,243 - HEAT**	**df**	**F**	**p**
Genotype	1,14	21.6	<0.05
Treatment	1,14	51.3	<0.05
Treatment × genotype	1,14	10.5	<0.05

**Table 7:** Effects of CL-316,243 on respiratory exchange ratio in WT and UCP-1 KO mice – statistical results.

0.04 mg/kg CL-316,243 - RER	df	F	p
Genotype	1,14	0.3	n.s.
Treatment	1,14	33.2	<0.05
Treatment × genotype	1,14	1.8	n.s.
**0.2 mg/kg CL-316,243 - RER**	**df**	**F**	**p**
Genotype	1,14	0.9	n.s.
Treatment	1,14	3.4	n.s.
Treatment × genotype	1,14	2.5	n.s.
**1 mg/kg CL-316,243 - RER**	**df**	**F**	**p**
Genotype	1,14	0.1	n.s
Treatment	1,14	88.6	<0.05
Treatment × genotype	1,14	2.3	n.s

**Table 8:** Effects of CL-316,243 3 on VO_2_ in WT and UCP-1 KO mice – statistical results.

0.04 mg/kg CL-316,243 – VO_2_	df	F	p
Genotype	1,14	7.3	<0.05
Treatment	1,14	8.1	<0.05
Treatment × genotype	1,14	0.7	n.s.
**0.2 mg/kg CL-316,243 - VO**_**2**_	**df**	**F**	**p**
Genotype	1,14	4.2	n.s.
Treatment	1,14	30.7	<0.05
Treatment × genotype	1,14	12.3	<0.05
**1 mg/kg CL-316,243 - VO**_**2**_	**df**	**F**	**p**
Genotype	1,14	9.2	<0.05
Treatment	1,14	70.7	<0.05
Treatment × genotype	1,14	13.0	<0.05

**Table 9:** Effects of CL-316,243 on food intake in WT and UCP-1 KO mice – statistical results.

0.04 mg/kg CL-316,243 - Food Intake	df	F	p
Genotype	1,14	8.8	<0.05
Treatment	1,14	98.9	<0.05
Treatment × genotype	1,14	10.6	<0.05
**0.2 mg/kg CL-316,243 - Food Intake**	**df**	**F**	**p**
Genotype	1,14	2.0	n.s.
Treatment	1,14	56.4	<0.05
Treatment × genotype	1,14	1.4	n.s
**1 mg/kg CL-316,243 – Food Intake**	**df**	**F**	**p**
Genotype	1,14	1.8	n.s
Treatment	1,14	60.3	<0.05
Treatment × genotype	1,14	5.3	<0.05

### The effects of CL-316,243 on sleep-wake activity and body temperature in control and mice with iBAT sensory afferents denervation

BAT-CAPS mice had significantly lower iBAT CGRP levels compared to controls (BAT-CAPS: 4.05 ± 0.21 pg/mg vs. BAT-VEH 12.30 ± 1.23 pg/mg; p < 0.001, Student’s t-test), indicating a ~70% decrease and confirming the efficiency of intra-BAT capsaicin treatment to reduce the sensory innervation of the organ.

**Figure 8:**
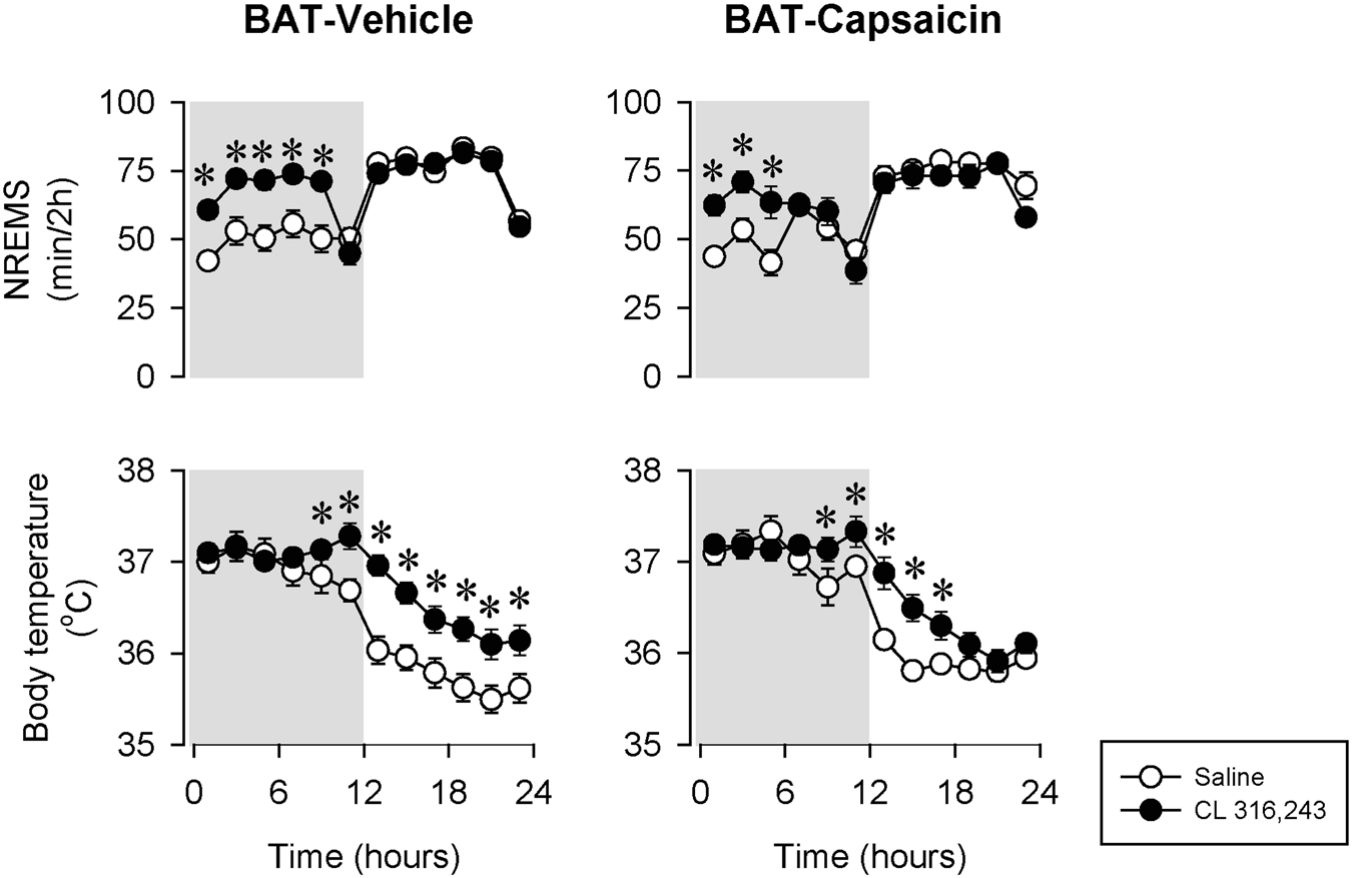
The effects of the selective β3-AR agonist CL-316,243 on NREMS and body temperature in mice with the chemical deafferentation of the intra-scapular BAT pads (BAT-Capsaicin; black symbols) and control mice (BAT-Vehicle; open symbols). See Fig. 1 for details.

To test the involvement of BAT sensory innervation in β3-AR-induced sleep, 0.2 mg/kg CL-316,243 was injected to intra-BAT CAPS-treated mice (Fig. 8). Sleep responses to CL-316,243 were attenuated by ~50% in intra-BAT CAPS-treated mice as compared to vehicle-injected controls. NREMS increase in the first 12-h after CL injection in the control mice was 92.4 ± 11 min while it was 57.3 ± 11.6 min in BAT-CAPS animals (p < 0.05, Student’s t-test). The effects on body temperature tended to be shorter lasting in BAT-CAPS animals, but did not differ significantly from those in BAT-VEH mice.

## Discussion

Activation of β3-ARs brings about an array of metabolic changes characterized by suppression of feeding, elevated energy expenditure, increased lipolysis, remodeling of WAT, thermogenic activation of BAT and the proliferation of brite, UCP-1 positive, adipocytes in the WAT. We report here that these profound metabolic effects are also accompanied by robust increases in NREMS in mice. Systemic injection of CL-316,243, a selective β3-AR agonist, produced rapid and marked increases in NREMS, increased body temperature and energy expenditure, decreased RER and suppressed feeding. The effects were abolished or attenuated in UCP-1 KO mice indicating that intact thermogenesis by BAT is required for the somnogenic and metabolic effects of β3-AR activation. Further, sensory denervation of the iBAT suppressed CL-316,243-induced sleep indicating the involvement of BAT afferents in the somnogenic response.

The effects of β3-AR activation on sleep have been addressed in two previous studies, but significant limitations of those experiments prevent the valid interpretation of the findings. In one study, light onset injection of 3 and 10 mg/kg CL-316,243 suppressed REMS in rats, while 0.1 mg/kg was without significant effect^18^. Unfortunately, the effects on NREMS were not reported in that paper. Also, the CL-316,243 doses were 10–100-fold higher that in the present experiments thus non-specific effects of the drug were more likely to manifest. In another study, high doses of SR58611A, a brain-penetrant β3-AR agonist, suppressed REMS when injected during daytime in rats^19^. In that study, sleep was recorded only for 6 h after treatments, and vigilance states were determined by simple spectral analysis of the EEG. While spectral components of the EEG show correlation with vigilance states, spectral analysis by itself is insufficient to reliably determine sleep-wake states. In our experiments, we did not observe any effect on REMS. The differences between our findings and previous reports may be due to the fact that we injected CL-316,243 at dark onset, when spontaneous REMS is minimal in mice thus further suppression is difficult to achieve. Alternatively, there could be a species specific difference in the REMS responses between rats and mice.

The best characterized and most abundant expression sites for β3-AR are the brown and white adipose tissues^4, 5^. Both adipose tissues are sources of peripheral, sleep-inducing signals. For example, WAT is a major source of leptin, interleukin 6 (IL-6), and tumor necrosis factor-α (TNFα)^20–22^, hormones with potent sleep-promoting activities^23–25^. Also, we have previously reported that sleep loss activates the thermogenic machinery in BAT and BAT thermogenesis is required for the full expression of homeostatic sleep rebound after sleep loss^13^. Since BAT abundantly expresses β3-ARs and β3-AR stimulation activates BAT thermogenesis^8–10^, we tested if BAT thermogenesis is a contributing factor in CL-316,243-induced sleep. The thermogenic capacity of BAT is conferred by the presence of UCP-1, a mitochondrial proton carrier^26^. Systemic injection of CL-316,243 stimulates BAT thermogenesis and increases energy expenditure. These effects are abolished in UCP-1 KO mice indicating that BAT is a main effector of the metabolic actions of CL-316,243^27–29^. The NREMS-promoting effects of 0.04 mg/kg CL-316,243 were completely abolished in UCP-1 KO mice, while the somnogenic effects of the two higher doses were attenuated by ~50–70%. These findings indicate that intact BAT thermogenesis a key factor in CL-316,243-induced sleep.

There are several possible mechanisms that may mediate the effects of activated BAT on sleep. One, BAT is a main effector organ for non-shivering thermogenesis and BAT activation may lead to increased body temperature^3^. Elevated body temperature is often associated with increased NREMS, e.g., during the exposure to warm ambient temperature^30^ or after the injection of pyrogenic cytokines, such as IL-1β and TNFα^25, 31, 32^. It is unlikely, however, that CL-316,243-induced sleep is due to changes in core body temperature as indicated by the clear dissociation between the time courses of the sleep and temperature effects. The sleep-promoting effects of CL-316,243 were immediate; in the first hour after the β3-AR agonist treatment, NREMS was already elevated by ~50%. Body temperature, however, became elevated only in hours 5–6 after the treatment. Two, BAT is innervated by thermo-sensitive afferents^17^. Signaling via these afferents is key to sleep loss-induced rebound sleep^13^. In the present study, lesion of the capsaicin-sensitive BAT afferents attenuated CL-316,243-induced sleep about the same degree as the lack of thermogenic protein, UCP-1. This strongly suggests that neuronal signaling from the activated BAT is a major component of BAT-induced sleep.

While the somnogenic effects of the lowest dose of CL-316,243 were completely abolished in UCP-1 KO mice, the effects of higher doses were not completely suppressed in UCP-1 KO or in BAT deafferented animals. This suggests that in high doses, CL-316,243 also activates somnogenic mechanisms that are not related to BAT thermogenesis. It is unlikely that the BAT-independent component of CL-316,243-induced sleep is due to the activation of central β3-ARs. β3-AR expression in the brain is low^33^ and central receptors are not reached by systemically administered CL-316,243 due to its inability to cross the blood-brain barrier^34^. Similarly to the sleep-inducing effects, the stimulatory effects of 1 mg/kg CL-316,243 on VO_2_ were not completely abolished in UCP-1 KO animals. This strongly suggests that relatively high doses of the β3-AR agonist activate targets, e.g., the white adipose tissue, where heat production is independent of UCP-1 expression.

CL-316,243 induces the production and release of several humoral factors from peripheral tissues that are known to induce NREMS. CL-316,243 induces 2–4-fold increases in circulating fatty acid levels with a latency of only a few minutes^8, 9, 35^. Various forms of fatty acids are known to induce^36–38^, while inhibition of fatty acid synthase to suppresses sleep^39^. Also, fatty acids are activators of UCP-1 in brown adipocytes and BAT thermogenesis^40, 41^ thus fatty acids may play a role in both the BAT-dependent as well as the BAT- independent components of the sleep-inducing effects of CL-316,243. A role for fatty acids in mediating the effects of CL-316,243 on metabolism in mice was previously proposed^42^. Further, β3-AR activation stimulates IL-6^42–45^ and insulin secretion^8, 9, 46^ resulting in a 6 and 50–140 fold increase in their respective plasma levels. Both insulin and IL-6 induce sleep when administered in bolus^24, 47–49^. Finally, bolus injection of CL-316,243 causes a 2–15 fold increase in TNFα mRNA expression in the WAT^45, 50, 51^. TNF is one of the best characterized NREMS-inducing hormone/adipokine^52^. It is possible that the combination of these, and potentially other, sleep-promoting molecules is responsible for the UCP-1-independent component of CL-316,243-induced NREMS.

The two-process model is a widely accepted model of sleep regulation. It describes sleep as the function of a circadian and a homeostatic factor^53^. The circadian component is driven by the biological clock in the suprachiasmatic nucleus. The homeostatic component is determined by the duration of prior wakefulness; prolonged wakefulness, such as occurring during sleep deprivation, is followed by longer and more intense/deeper NREMS periods. SWA of the EEG is commonly regarded as the indicator of NREMS intensity and the reflection of the activity of the homeostatic process^53^. EEG SWA is elevated after sleep loss indicating an increased homeostatic sleep pressure due to unfulfilled sleep need. In our studies, increased NREMS after β3-AR agonist treatment was accompanied by significant decreases in SWA. This decrease, however, was not specific to the SWA (0.5–4.5 Hz) range, but it was also present in the higher frequencies. Therefore, it is likely that suppressed SWA in response to CL-316,243 is not a reflection of decreased NREMS intensity, but it is part of a broader suppressive effect on sleep EEG.

Our findings confirm previous reports that systemic bolus injection of CL-316,243 leads to elevated body temperature, energy expenditure and suppressed feeding^8–10^. These effects were significantly attenuated in UCP-1 KO mice, but, similarly to the sleep-promoting actions, were not completely abolished. This suggests that thermogenic activation of BAT is a major component in these effects, but other, UCP-1-independent mechanisms also play a role. These mechanisms could be shared with the UCP-1-independent sleep-inducing machinery, as several of the bioactive molecules, such as IL-6 and TNFα, which are released in response to β3-AR, not only produce sleep but also elicit fever and anorexia^54^. Our findings give support to the idea that WAT directly or indirectly participates in the overall thermogenic response to acute β3-AR stimulation and that a component of thermogenesis is UCP-1 independent^27^.

In summary, we demonstrated that activation of β3-ARs by systemic injection of a selective ligand elicits robust sleep increases, as well as coordinated metabolic responses. Major component of these actions requires the intact thermogenic capacity of the brown adipose tissue. These findings confirm the importance of systemic signals arising from metabolic organs in sleep regulation and gives support to the notion that regulation of sleep and metabolism is highly integrated and are consistent with our multi-process model that in addition to the homeostatic and circadian processes, other, mainly metabolically driven process(es) are integral part of the complex sleep-regulatory mechanism.

## Methods

### Animals and surgery

Male, 5–6 months old WT C57BL/6 and congenic UCP-1 KO mice were used in the experiments. Breeding pairs of the UCP-1 KO animals were obtained from Dr. Leslie P. Kozak of Pennington Research Institute (Baton Rouge, LA) and were further bred at Washington State University. Each mouse used in the experiments was genotyped (Transnetyx, Cordova, TN). All animal work was conducted in accordance with the recommendations in the Guide for the Care and Use of Laboratory Animals of the National Institutes of Health. The protocol was approved by the Committee on the Ethics of Animal Experiments of the Washington State University.

The body weight of the WT and UCP-1 KO mice at the time of surgery was 31.3 ± 0.9 g and 31.2 ± 0.4 g, respectively. All surgical procedures were performed with the mice under ketamine-xylazine anesthesia (87 and 13 mg/kg, respectively). For polysomnography recordings, the animals were implanted with cortical EEG electrodes, placed over the frontal and parietal cortices, and nuchal electromyographic (EMG) electrodes. The EEG and EMG electrodes were anchored to the skull with dental cement. Telemetry transmitters were implanted intraperitoneally for body temperature and motor activity recordings. To lesion the small-diameter sensory afferents arising from the iBAT selectively, anesthetized mice were injected with 20 microinjections (1 µl/injection) of 20 µg/µl of capsaicin into the iBAT (BAT-CAPS, n = 7, BW = 37.9 ± 1.1 g) to cover the full extent of both iBAT pads^16^. Mice in the control group were injected with vehicle (10% ethanol, 10% Tween 80, in isotonic NaCl, BAT-VEH, n = 8, BW = 34.7 ± 1.7 g). These animals were instrumented for sleep recordings 3 weeks after BAT treatments. All mice were allowed to recover from surgery for at least 10 days before any experimental manipulation started. During the recovery and experimental periods, mice were housed individually in a sound-attenuated environmental chamber at controlled light–dark cycles (12–12 h, lights on: 4 am) at 30 ± 1 °C ambient temperature. Food (regular lab chow; Harlan Teklad, Product No. 8460) and water were available *ad libitum* throughout the experiments.

### Data collection and analyses

The animals were tethered to commutators, which were further routed to Grass Model 15 Neurodata amplifier system (Grass Instrument Division of Astro-Med, Inc., West Warwick, RI). The amplified EEG and EMG signals were digitized at 256 Hz and recorded by computer. The high-pass and low-pass filters for EEG signals were 0.5 and 30.0 Hz, respectively. The EMG signals were filtered with low and high cut-off frequencies at 100 and 10,000 Hz, respectively. The outputs from the 12A5 amplifiers were fed into an analog-to-digital converter and collected by computer using SleepWave software (Biosoft Studio, Hersey, PA). Sleep-wake states were scored visually off-line in 10-s segments. The vigilance states were defined as NREMS, REMS and wakefulness (W) according to standard criteria as described previously^39^. Time spent in W, NREMS and REMS was calculated in 2-, 4-, 6-, 12- and 24-h blocks. EEG power data from each artifact free 10-s segment were subjected to off-line spectral analysis by fast Fourier transformation. EEG power data in the range of 0.5 to 4.0 Hz during NREMS were used to compute EEG SWA. EEG SWA data were normalized for each animal by using the average EEG SWA across 24 h on the baseline day as 100. The results were further averaged in 2-h bins.

### Telemetry recordings

For the sleep experiments, core body temperature was also recorded by MiniMitter telemetry system (Philips Respironics, Bend, OR) using VitalView software. Temperature values were collected every 1 min throughout the experiment and were averaged over 2-h time blocks.

### Experimental procedures

#### Experiment 1: The effects of CL-316,243 on sleep-wake activity, body temperature and metabolic parameters in WT and UCP-1 KO mice

Mice (n = 7 for WT and n = 6 for UCP-1 KO mice) received subcutaneous injections of CL-316,243 (Sigma-Aldrich) or saline 10–15 min before the onset of the dark period. The three doses of CL-316,243 were of 0.04, 0.2, and 1 mg/kg. All the mice were injected with all three doses of CL-316,243 with at least 7 days between the CL-316,243 injection days.

In a separate group of mice (n = 8 for both WT and UCP-1 KO mice) oxygen consumption (VO_2_, ml/kg/h) and carbon dioxide production (VCO_2_, ml/kg/h) were measured with indirect calorimetry (Oxymax System, Columbus Instruments, Columbus, OH). Twenty-four hour food intake (g/kg/h) was also recorded. All three measurements were taken every 10 min and the data were collapsed into 24-h bins. Respiratory exchange ratio (RER; VCO_2_/VO_2_) was calculated. The animals were habituated to the calorimetry cages for three days before the experiments. On the fourth day mice were injected with saline, on the fifth day they received one of the three doses of CL-316,243.

#### Experiment 2: The effects of CL-316,243 on sleep-wake activity and body temperature in control and mice with sensory afferents denervation

To determine if somnogenic signals arising from BAT in response to β3-AR stimulation are mediated by the sensory afferents of BAT, we tested the effects of CL-316,243 in C57/BL6 mice with the chemical deafferentation of the iBAT. Both BAT-VEH (n = 8) and BAT-CAPS (n = 7) mice were injected with 0.2 mg/kg CL-316,243 10–15 min before the onset of the dark phase. Each mouse was recorded for 24 hours starting after injections. To verify that intra-BAT injection of capsaicin did not cause the degeneration of small-diameter sensory nerves systemically, eye-wiping test was performed using 0.01% capsaicin solution^55^. All BAT-CAPS mice showed positive eye-wiping test indicating intact nociceptive innervation of the cornea, thus capsaicin injections remained localized in the BAT. At the end of the experiment, iBATs were removed, weighed, and stored at −80 °C.

Verification of iBAT sensory denervation. iBAT samples were homogenized in 2 N acetic acid. Calcitonin gene-related peptide (CGRP) levels in iBAT were measured using a commercially available EIA kit (Bertin Pharma/SPI-Bio, Montigny-le-Bretonneux, France) according to the manufacturer’s instructions. For the CGRP assays, the correlation coefficient was 99.8%.

### Statistics

The amounts of NREMS, REMS, wakefulness, as well as EEG SWA, body temperature, VO_2_, and RER data in Experiment 1 were calculated in 2-h blocks. Three-way mixed ANOVA was performed separately for each dose of CL-316,243 across 24 h (independent measure: genotype, repeated measures: time and treatment). Daily food intake, VCO_2_, VO_2_ and RER were analyzed by using two-way mixed ANOVA (independent measure: genotype, repeated measure: treatment). When ANOVA indicated significant effects, *post hoc* paired t-tests (for repeated measures) or Student’s t-tests (for independent measures) were performed for all experiments.
